# Cholera Risk: A Machine Learning Approach Applied to Essential Climate Variables

**DOI:** 10.3390/ijerph17249378

**Published:** 2020-12-15

**Authors:** Amy Marie Campbell, Marie-Fanny Racault, Stephen Goult, Angus Laurenson

**Affiliations:** 1 European Space Agency, Climate Office, ECSAT, Harwell OX11 0FD, UK; climate.office@esa.int or; 2 Plymouth Marine Laboratory, Prospect Place, The Hoe, Plymouth PL1 3DH, UK; stgo@pml.ac.uk (S.G.); anla@pml.ac.uk (A.L.); 3 National Centre For Earth Observation, PML, Plymouth PL1 3DH, UK

**Keywords:** cholera, coastal environment, climate, remote sensing, essential climate variables, machine learning, AI, random forest

## Abstract

Oceanic and coastal ecosystems have undergone complex environmental changes in recent years, amid a context of climate change. These changes are also reflected in the dynamics of water-borne diseases as some of the causative agents of these illnesses are ubiquitous in the aquatic environment and their survival rates are impacted by changes in climatic conditions. Previous studies have established strong relationships between essential climate variables and the coastal distribution and seasonal dynamics of the bacteria *Vibrio cholerae*, pathogenic types of which are responsible for human cholera disease. In this study we provide a novel exploration of the potential of a machine learning approach to forecast environmental cholera risk in coastal India, home to more than 200 million inhabitants, utilising atmospheric, terrestrial and oceanic satellite-derived essential climate variables. A Random Forest classifier model is developed, trained and tested on a cholera outbreak dataset over the period 2010–2018 for districts along coastal India. The random forest classifier model has an Accuracy of 0.99, an F1 Score of 0.942 and a Sensitivity score of 0.895, meaning that 89.5% of outbreaks are correctly identified. Spatio-temporal patterns emerged in terms of the model’s performance based on seasons and coastal locations. Further analysis of the specific contribution of each Essential Climate Variable to the model outputs shows that chlorophyll-a concentration, sea surface salinity and land surface temperature are the strongest predictors of the cholera outbreaks in the dataset used. The study reveals promising potential of the use of random forest classifiers and remotely-sensed essential climate variables for the development of environmental cholera-risk applications. Further exploration of the present random forest model and associated essential climate variables is encouraged on cholera surveillance datasets in other coastal areas affected by the disease to determine the model’s transferability potential and applicative value for cholera forecasting systems.

## 1. Introduction

The bacteria *Vibrio cholerae* are a native constituent of aquatic environments [1], existing generally in brackish, coastal and oceanic waters as free floating bacterioplankton or attached to non-living particles and living hosts [2,3,4]. These bacteria are heterotrophic and play key roles in aquatic ecosystems, through host-pathogen and predatory-prey interactions, as well as in biogeochemical cycling [5,6]. The bacteria *V. cholerae* become pathogenic by acquiring virulence factors, including toxin co-regulated pillus, cholera toxin, and motility that give them the ability to infect and cause symptoms in the host, including in human.

Pathogenic *V. cholerae* bacteria (serotypes O1 and O139) are the aetiological agent responsible for human cholera disease [7], causing intestinal infection, acute diarrhoea and dehydration, which can be fatal within hours if left untreated. Humans can be exposed to the pathogenic bacterium through consumption and drinking of contaminated seafood and water respectively, and during recreational activities in contaminated waters. While a large number of cholera cases may go non- or mis-reported, modelling studies estimated 2.9 million cases annually, with around 1.3 billion people currently at risk of being infected with cholera [8]. Over half of the cholera cases reported by the World Health Organisation between 2010 and 2016 came from countries bordering the northern Indian Ocean [9], which provided the region for this study. The densely populated coastal zones of Bangladesh and India, with approximately 35 and 200 million people respectively [10,11], have a long history of cholera outbreaks and remain among the high-risk endemic countries [8]. Although the burden of cholera is likely to be underestimated in these countries, nationwide surveillance programmes such as the International Centre for Diarrhoeal Disease Research in Bangladesh (ICDDR, B) and the Integrated Disease Surveillance Programme (IDSP) in India, carry out event-based surveillance of cholera outbreaks which provides consistent datasets to explore trends in cholera outbreaks.

Cholera-risk mitigation includes increasing preparedness and resilience through surveillance programmes, forecast model developments, capacity building, including education and citizen science projects [12,13], as well as training of health and environmental officials so they can respond accordingly to reduce the emergent risks [14]. Cholera-risk studies distinguish between potential mitigation strategies for epidemic cholera-generally sporadic and located further inland- and endemic cholera- with recurring incidence for consecutive years in coastal locations [15]. To date, it is the latter that has provided the most scope to study periodic oscillations in cholera disease incidence [16] which then can be used to inform research on environmental cholera risk.

The coastal areas of India exhibit extreme environmental disturbances with the Summer monsoon seasonally-reversing winds which are coupled with a strong annual cycle of precipitation (wet summer and dry winter) and cause large seasonal variations of inflows from the Ganges and other rivers [17]. Additionally, the environmental conditions in coastal areas are characterised by strong inter-annual variability with basin-scale influence of global and regional climate modes of variability, such as El Niño and Indian Ocean Dipole respectively [18,19,20,21]. These characteristics provide plenty of scope to assess whether satellite-derived climate records can extract these patterns of climate variability and explore the relationships with the seasonal and spatial dynamics of cholera outbreaks.

To our knowledge, relationships with cholera outbreaks have so far been demonstrated with seven essential climate variables [22], including sea surface temperature [16,23,24,25,26,27]; salinity and saltwater intrusions [16,25,27,28,29]; plankton and chlorophyll-a concentration [1,4,16,30]; sea level/height [24,26,31]; precipitation [5,26,32]; land surface temperatures [15,32,33]; and soil moisture [16]. A number of these studies used field or laboratory experiments with *V. cholerae* while others have taken an epidemiological approach to explore relationships with actual cholera incidence or outbreaks.

Identifying seasonal drivers of cholera can be difficult, due to the covariation of different environmental variables [29] which leads to confounding processes where ECVs may exacerbate or minimize the impact of another in a series of complex interactions, as highlighted in an example by [32]. Using machine learning can provide a solution to these issues as the algorithms are capable of developing and recognizing patterns and complex relationships across large datasets, and this capacity offers particular value when these relationships are either unknown or suspected. To our knowledge, only a few previous studies have explored the use of machine learning techniques for cholera, in terms of serological markers [34] or environmental drivers [35].

The present study investigates a novel use of machine learning analyses of satellite-derived ECVs to predict environmental cholera risk in coastal districts in India during the period of 2010–2018. In particular, we explore the use of random forest classifiers and evaluate their application potential to analyze, in combination, seven satellite-derived ECVs. Furthermore, to our knowledge, we investigate for the first time, the use of remotely-sensed sea surface salinity in an application of machine learning analyses of a combination of ECVs to detect the risk of cholera outbreaks. We perform a sensitivity analysis of different machine learning techniques using accuracy, F1 score and sensitivity metrics and assess the predictive value of each ECV included in the analysis. Finally, we discuss the potential of machine learning analyses of satellite-derived ECVs for the development of outbreak monitoring systems in coastal regions affected by cholera.

## 2. Materials and Methods

### 2.1. Surveillance Data of Cholera Outbreaks

Surveillance data of cholera outbreaks at the district level in India over the period July 2009 to December 2019 were collected from publicly available weekly epidemiological reports published in Portable Document Format (PDF) by the Integrated Disease Surveillance Programme of India (IDSP) [36]. Each report describes the incidences of communicable diseases for a given week with detailed information per state and district on the disease, number of cases, number of deaths, start date, reporting date, current status and comments related to each outbreak. First, the PDF document of each report was converted into plain text using open-source PDF-to-text utility (www.xpdfreader.com). Then, a Python script utilizing the module for regular expressions was used to extract the tables containing data on cholera outbreaks. In the IDSP reports, post-2016 cholera outbreaks were given a unique ID code by the IDSP, which was used to delineate the outbreaks from one another, whilst pre-2016 outbreaks had to be first located by matching their date and then fully captured by incrementally working outwards from the text, using regular expressions to match the data values. Further processing using regular expressions and fuzzy text matching was carried out to identify and correct misspellings, homogenize the format of dates, and identifying anomalous reads such as dates outside of the valid range and the largest number of cases and deaths. Then the cholera data records were manually checked against each individual report in the original PDF documents. In addition, all extracted outbreak data were summed per state and per year, and validated against the total number of outbreaks per state per year which were published by IDSP for the years 2011–2015. A total of 630 cholera outbreaks were identified across all states in India. A shapefile was generated for each cholera outbreak describing the geographic area at district and state levels to enable comparison between the clinical incidence data and ECVs.

The weekly cholera data records were aggregated by month (based on the month of the outbreak ‘start date’) and by district, based on the Level 2 administrative zones for India provided in shapefile format by the Database of Global Administrative Areas [37]. Cholera outbreaks were converted into a binary data format with a value of one indicating outbreak in a particular month and district, and a value of zero indicating no outbreak. The cholera outbreak binary data were then used as an effective variable type for machine learning applications.

### 2.2. Essential Climate Variables

A total of six ECV datasets were obtained from the ESA CCI programme, which provides climate-quality controlled data from Earth Observation satellites [38] (Table 1). In addition, the satellite altimetry data product produced by Ssalto/Duacs and distributed by AVISO was downloaded for the period 2016–2018 to complement the CCI Sea Level data available over the period 2010–2015. Finally, the ECV data of total precipitation of the European Reanalysis Interim (ERAI) synoptic monthly means were obtained from the European Centre for Medium-Range Weather Forecasts (Table 1). All datasets were extracted and prepared in NetCDF format for the period 2010–2018 and bounding box region located between 0–40° N and between 100–60° E.

The ECV data were all re-sampled to a monthly resolution, taking the monthly averages from datasets with a higher temporal resolution. A monthly resolution allows for possible incubation periods of *V. cholerae* and accommodates potential lags between infection incidence and reporting. This resolution has also been used in previous studies, which have reported that the cholera-incidence response range to environmental variables within a week is generally insignificant [35]. The chlorophyll-a concentration dataset was converted to a logarithmic scale to accommodate the large range of values, spanning three orders of magnitude. All ECV data were then re-sampled at the district level. For terrestrial variables, a mean value was calculated over each district area. For oceanic variables, the ECV data were first extracted over the coastal area extending from district shoreline to one decimal degree offshore, and then a mean value was calculated for each coastal district area (hereafter ‘coastal’ district refers to districts that have a one-degree buffer that interacts with the coverage of oceanic ECV datasets). District and coastal zone areal means were computed utilizing the Python packages Rasterio, GeoPandas and xarray [48] for each time step to create a monthly time series for the period 2010–2018. This was then appended to the cholera data time series using Python Pandas [49] dataframe tools.

Lag effects were studied for both one and two months before the outbreak, as previous studies have found some environmental indicators to have lagged effects on cholera incidence [15,24,26,32]. Specifically, we generated lag datasets as follows: (1) we used monthly values from one month and two months prior to cholera incidence; (2) we calculated the rate of change between the previous two months; and (3) we computed a binary indicator based on whether the value one-month and two-month prior had been higher or lower than the current month when a cholera outbreak was reported.

### 2.3. Model Development

Our study used the Random Forest (RF) Classifier [50] implementation included in Scikit Learn [51]. This is an ensemble learning method that fits multiple decision tree classifiers and uses bootstrapping to output a decision averaged over these predictors, to improve prediction accuracy and reduce effects of over-fitting. This approach is consistent with previous literature investigating similar outbreak risk indexes which have demonstrated RF classifiers to be an appropriate method for the application to epidemiological data along with analyses of environmental factors, including for prediction of outbreaks of cholera disease (e.g., [35]), vector-borne diseases such as dengue (e.g., [52,53]) and malaria (e.g., [54]), and the avian influenza H5N1 zoonotic disease outbreaks (e.g., [55]).

**Figure 1:**
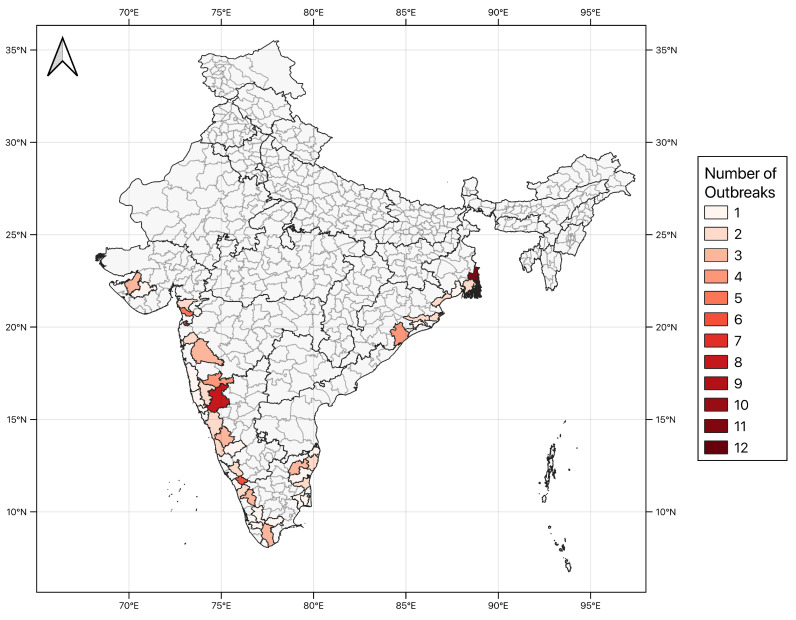
Number of cholera outbreaks reported in the weekly epidemiological reports published by the Integrated Disease Surveillance Programme of India (IDSP) [36] during the period January 2010 to December 2018 for the 40 coastal districts of India selected in this study. Only the districts reporting cholera incidence data and for which all seven available Essential Climate Variable (ECV) datasets are shown. The study period was chosen based on the availability of cholera data and ECV time-series data (please see Table 1).

The RF classifier was trained and tested to return a binary output (1 for an outbreak predicted, 0 for no outbreak), and the optimum number of estimators were also assessed. The study analyses focused on districts with a direct coastal border and districts located within ∼100 km of the coast. The chosen study period was from January 2010 to December 2018 based on the availability of cholera data and time-series data for all ECVs (Table 1). Furthermore, only the districts reporting cholera incidence data and for which all seven ECV datasets were available have been retained, and in the end a total of 40 districts were included in the analyses (Figure 1). These selection criteria allowed us to ensure that a consistent number of ECVs were taken into account in the machine learning analyses. Numerical features were generated for two categorical variables using integer encoding: (1) seasons, based on the definition from the India Meteorological Department (mausam.imd.gov.in) for winter (JF: Jan.–Feb.), pre-monsoon (MAM: Mar.-Apr.–May), monsoon (JJAS: Jun.–Jul.–Aug.–Sep.) and post-monsoon (OND: Oct.–Nov.–Dec.); and (2) coastal location (East or West). The latter feature was chosen based on previous reports showing large variability in hydroclimactic responses to the monsoon between eastern and western coasts of India [56].

The RF model input cholera dataset was largely imbalanced, with 77 outbreak and 8504 non-outbreak data points found in the monthly time series for 40 coastal districts. As such, the model accuracy would largely reflect the underlying class distribution. Dataset imbalances have previously been identified as a key challenge accompanying machine learning [57], including applications for cholera analysis [35]. Previous studies have attempted to overcome the issue of imbalanced datasets through under-sampling (decreasing samples of majority classes) or over-sampling (increasing samples of minority class). In this study, we utilized the Synthetic Minority Oversampling Technique (SMOTE) in the pre-processing stage, which allowed us to generate new examples of the minority class based on lines drawn between random existing examples in the feature space using k-nearest neighbours [58]. Different imbalance ratios were considered using a sensitivity analysis; and generally, the more balanced the dataset, the higher the accuracy results. However, assuming a 1:1 ratio of outbreaks against non-outbreaks would be unrealistic with real-world data, and a ratio of 1:10 was used. The latter ratio permitted a balance between machine learning requirements and realistic applications to cholera outbreaks analysis. Finally, the model input dataset contained a total of 850 outbreak and 8504 non-outbreak data points. The input dataset was split into a training dataset, randomly selecting 70% of the data, and a test dataset (so-called ’unseen dataset’) with the remaining 30%. Further splitting of the latter into a validation dataset and a separate test dataset might have been useful to explore the model accuracy with test data independent from the model development validation dataset, however, the limited number of cholera outbreak datapoints available in our nine-year study period meant this was unfeasible. Specifically, a 70:20:10 split between the training, validation and test datasets respectively would have generated a test dataset containing only ∼85 outbreaks spread over a limited number of districts which would have been insufficient to test and explore the applicability of the model across the large spatial range of coastal districts in India. To assuage this issue, we used a 70% training and 30% test split, with the training dataset containing 605 outbreaks over 40 coastal districts and the test dataset containing 245 outbreaks over 36 coastal districts.

The RF Model was refined based on systematic testing of optimum features and parameters, and the recorded impact these had on performance metrics which were evaluated using the test dataset. Specifically, to refine the model, we tested approximately 30 different models including different combinations of ECVs and their respective binary lagged values, as well as the inclusion of the season and location encoders. The inclusion or removal of variables and encoders were implemented based on their contributions to improve cholera outbreak predictions of the test dataset. The models’ predictions were assessed using the metrics of accuracy (1), which takes all components into account including true positives (TP), true negatives (TN), false positives (FP) and false negatives (FN):

(1) $$Accuracy=\frac{TP+TN}{TP+TN+FP+FN};$$

the F1 Score (2), providing a harmonic mean of both precision and sensitivity, with its common usage in machine learning studies increasing comparability to other study’s model performance:

(2) $$F_{1}\;Score=\frac{2TP}{2TP+FP+FN};$$

and importantly, the sensitivity (3), which provides an estimate of the model performance in terms of how many positives (cholera outbreaks) are correctly identified:

(3) $$Sensitivity=\frac{TP}{TP+FN}.$$

**Figure A1:**
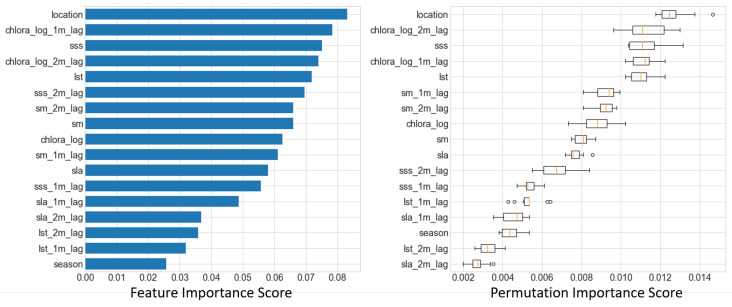
Variables included in the Random Forest classifier. The variables are ranked in terms of feature importance (**left**) and permutation importance (**right**).

The full list of variables and filtered selection is described in Appendix A Figure A1. The best-performing parameters and features were explored using collinearity analysis based on Spearman’s Rank correlation, generating dendrograms and applying clustering thresholds to select independent features. Statistical significance is calculated using the Python SciPy’s ‘spearmanr’ function [59], which returns the exact two-tail probabilities critical p-value based on the null hypothesis that two sets of data are uncorrelated. In addition, to reduce the possible incidence of feature auto-correlation, we assessed the importance of each feature in the available input ECV data. The RF model produced an ensemble of trained estimators that were independently trained on random subsets of the available input features, providing a further step to help to reduce the incidence of auto-correlation.

**Figure A2:**
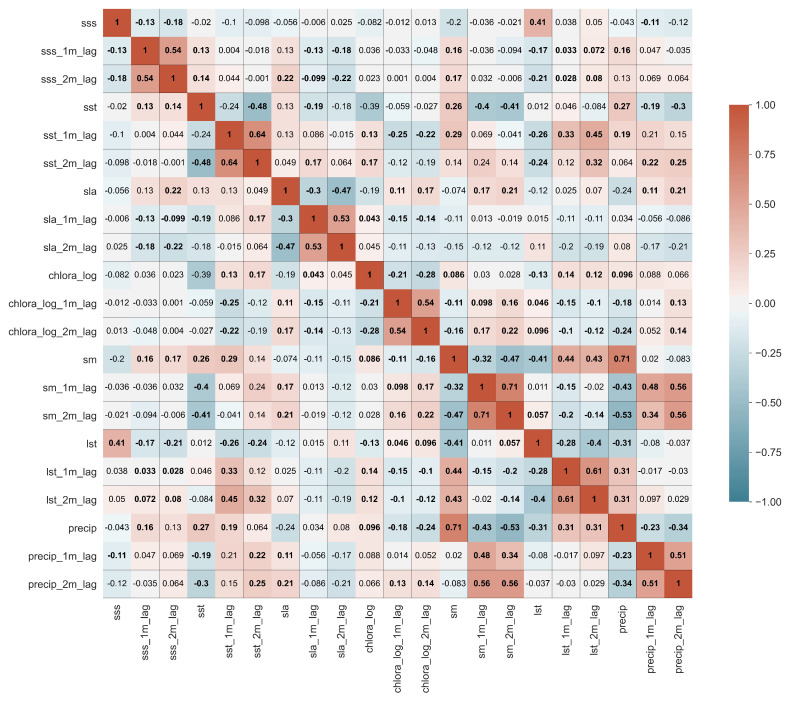
Spearman’s Rank Correlation Coefficient analyses for all essential climate variables and lagged values considered in the present study. Significant *p*-values (<0.05) are shown in bold. Statistical significance is calculated using a two-sided *p*-value where the null hypothesis is that the two sets of data are uncorrelated.

**Figure A3:**
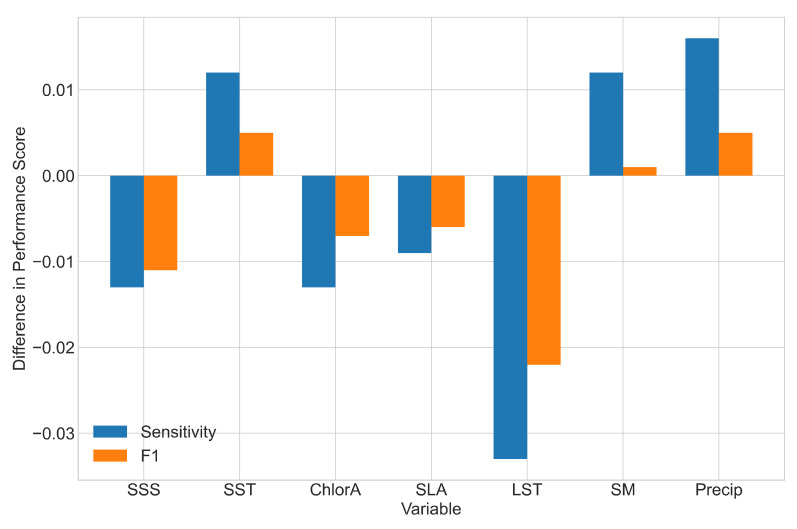
Effect of individual variable permutation on the model performance metrics. The performance assessments were carried out by omitting one variable (out of seven) and then the Sensitivity and F1 scores were estimated. The difference in the performance is calculated as the difference between the scores of the model run with all seven variables minus the model run with six variables. The encoders for seasonal and location were included in all model runs. The analyses were performed using the outbreak test data and the variables’ permutation was done for Surface Temperature (LST), Chlorophyll a Concentration (ChlorA), Sea Surface Salinity (SSS), Sea Surface Temperature (SST), Sea Level Anomalies (SLA) and Soil Moisture (SM) and total precipitation (Precip).

The variables of precipitation and soil moisture were found to be significantly correlated (Spearman’s Rank Correlation Coefficient = 0.71, *p*-value < 0.05, Appendix A Figure A2). Furthermore, systematic testing by removing combinations of ECVs and calculating variables’ permutation importance to find the optimum use of unique information for the model showed that dropping precipitation and sea surface temperature had the greatest increase on all three model performance metrics (Appendix A Figure A3).

The final model, which will be referred to as the RF Model, included five ECVs and their respective binary-lagged values, as well as the season and location encoders. The RF model results were evaluated using Confusion Matrices that visually display the classification labels against the true labels, and receiver operating characteristics curves that illustrate the diagnostic skill of the binary classifier. The contribution of the individual ECVs and their respective lagged values were also investigated through calculating permutation importance and running the model with only individual variables. In addition, the RF was tested on the unseen data for individual districts, and the performance metrics were obtained for sensitivity, F1 Score and accuracy to explore spatial trends in district-level performance.

## 3. Results

### 3.1. Random Forest Model Development

**Figure 2:**
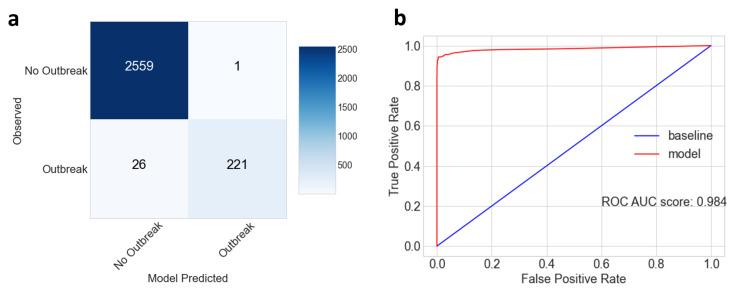
Model Results for all coastal districts in India. (**a**) Confusion matrix of the random forest model cholera outbreak prediction results on unseen test data. Clockwise from the top right corner, the cells show true negatives, false positives, true positives and false negatives for no-outbreak and outbreak between model results and observations. (**b**) Receiver operating characteristic (ROC) curve showing the diagnostic ability of the RF model binary classifier in red, compared to a baseline in blue (1:1 line corresponding to random prediction), with the area under curve (AUC) score displayed.

**Figure A4:**
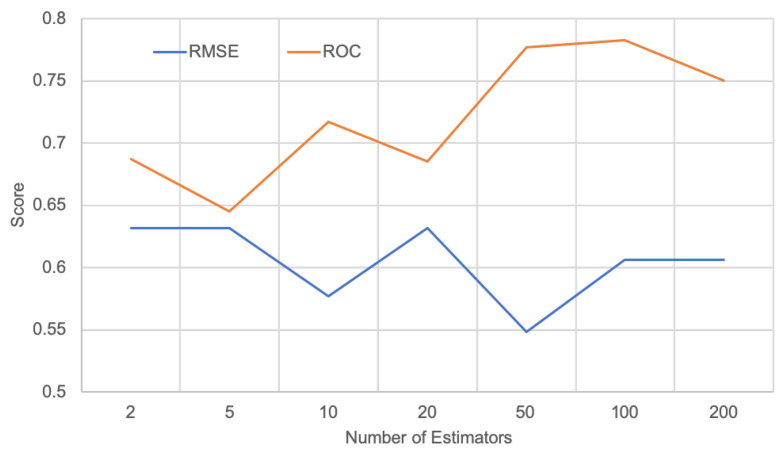
Exploration of the optimum number of estimators during the initial testing phase of the Random Forest model. The number of 50 estimators was chosen based on the model having the lowest Root Mean Square Error (RMSE) and the second-highest Receiver Operating Characteristic (ROC) accuracy.

Based on their positive contributions to the model performance, the final model included the season encoder, location encoder, five ECVs and their respective binary lag values. The optimum number of estimators was found to be 50 (see Appendix A Figure A4). When applied to the unseen test data for all coastal districts, the RF Model has an Accuracy of 0.99, an F1 Score of 0.942 and a sensitivity score of 0.895, meaning 89.5% of outbreaks were correctly identified. This translates to 221 of the 247 outbreaks in the unseen test data being predicted accurately, while 26 were missed, which equates to 11% of outbreaks (Figure 2a). One occasion saw a false positive of an outbreak being predicted when there were none recorded. However, as the remaining 2559 non-outbreak months were correctly identified, the model had high specificity. The Area Under Curve of the Receiving Operator Characteristic curve (Figure 2b) was estimated at 0.984 which shows a promising diagnostic ability to forecast occurrence or non-occurrence of outbreaks. This score represents the quality of the forecast, ranging from 0 to 1, where values of 0.9–1 can be considered excellent.

Previous studies have reported a distinct seasonal pattern for cholera outbreaks in regions where the disease is endemic [31,60,61]. In the present study, although the RF model was designed to predict cholera outbreaks in any season, by encoding the season as a feature, we observed that the model gave the highest sensitivity score of 0.933 in pre-monsoon outbreaks (Table 2). Pre-monsoon outbreaks made up 42.1% of all outbreaks in the test dataset. The model sensitivity scores for monsoon, post-monsoon and winter cholera outbreaks were 0.857, 0.886 and 0.868 respectively. These made up 28.3%, 14.2% and 15.4% of the test data outbreaks respectively.

### 3.2. Individual District Testing

**Figure 3:**
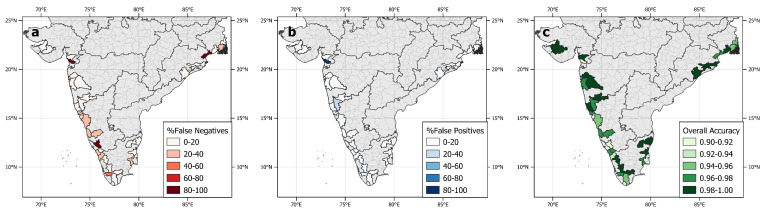
Performance metrics’ results of the random forest model when applied to unseen test data for individual districts in coastal India that reported cholera outbreaks: (**a**) Percent false negatives, (**b**) Percent false positives, and (**c**) Accuracy score. In all panels, coastal districts with no cholera outbreaks reported in the study period and/or non-coastal districts are shown in grey colour.

The RF model was tested on unseen data for 36 individual districts that contained reported outbreaks in the test data, to evaluate the spatial variation of model performance and its applicability to regions in India on a more localized scale (Figure 3). The model was able to correctly identify all of the outbreaks and non-outbreaks in districts that had multiple outbreaks in the test data, including: Purba Medinipur in West Bengal; Tiruvannamalai in Tamil Nadu; Ganjam, Kendrapara and Khorda in Odisha; Palakkad in Kerala; Sindhudurg and Sangli in Maharashtra; and Jamnagar in Gujarat. Some districts were associated with fewer outbreaks, and in some cases, their test datasets contained only one outbreak, such as in Balasore in Odisha and Navsari in Gujarat. In the latter districts, the one outbreak in their test datasets was missed by the model, resulting in these districts being labelled with false-negative results. Such instances generally reflected areas with fewer outbreaks. Conversely, the number of false positives remained low throughout the districts tested. These results suggest that the RF model is more difficult to assess on a per district level for representations of a single outbreak. However, when the test data contained multiple outbreaks at the district level, the RF model performed well with overall accuracy remaining above 0.9 at the individual district level.

At a state level, no link was found between the model sensitivity results and the percentage of outbreaks represented in the test dataset. The model sensitivity metrics remained above 0.80 in states containing more than ten outbreaks in the test dataset, including Odisha (sensitivity = 0.933), Kerala (sensitivity = 0.931), Maharashtra (sensitivity = 0.913), Tamil Nadu (sensitivity = 0.911), Karnataka (sensitivity = 0.846) and West Bengal (sensitivity = 0.875), which contained 6.1%, 29.1%, 9.3%, 18.2%, 21% and 13% of all outbreaks respectively. The states with fewer cholera outbreaks in the test dataset (<2% of total) showed mixed results, including Puducherry in which the only cholera outbreak was missed (leading to a model sensitivity = 0), Gujarat in which one of the three outbreaks was missed (sensitivity = 0.667), and Goa in which all four outbreaks were accurately detected (sensitivity = 1).

### 3.3. Sensitivity Analyses

#### 3.3.1. Individual ECVs

Permutation importance analysis showed that model accuracy decreased when ECVs and their respective lags were permuted, with the highest drops seen for land surface temperature followed by sea surface salinity and chlorophyll-a concentration (Appendix A Figure A3). In contrast, the model accuracy did not decrease when sea surface temperature, precipitation or soil moisture were permuted.

**Figure 4:**
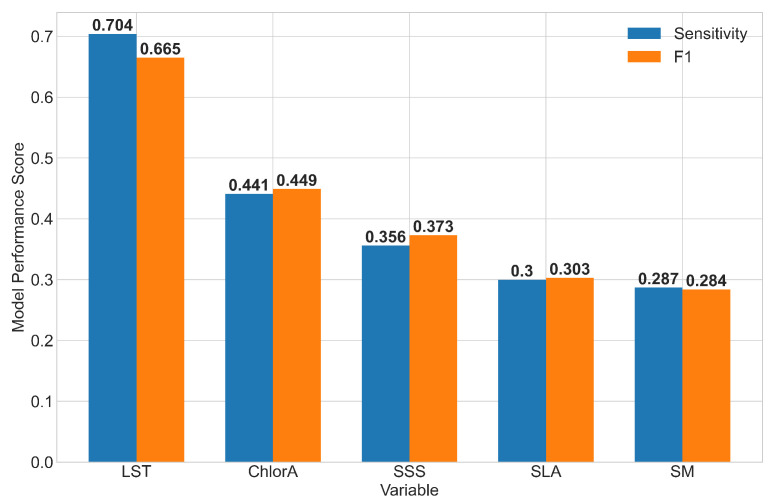
Performance metrics of the Random Forest model runs for each individual essential climate variable and its respective lags. The encoders for season and location were included in all model runs. The analyses were performed using the outbreak test data for the variables Land Surface Temperature (LST), Chlorophyll a Concentration (ChlorA), Sea Surface Salinity (SSS), Sea Level Anomalies (SLA) and Soil Moisture (SM).

Feature importance analysis of the ECVs included in the RF Model (Appendix A Figure A1) highlighted the top five ranked features respectively to be the location encoder, chlorophyll-a concentration (1-month binary lag prior to the outbreak month), sea surface salinity of the outbreak month, chlorophyll-a concentration (two-month binary lag prior to the outbreak month) and land surface temperature of the outbreak month. Additional models were also evaluated when using only an individual ECV and their respective lagged values, alongside season and location encoders, to explore individual variable contribution to the cholera outbreak forecast (Figure 4). The models were assessed using the same test dataset, and individually, the land surface temperature was the best performing variable (Sensitivity = 0.704, F1 Score = 0.665), followed by chlorophyll-a concentration, sea surface salinity, sea level anomalies and soil moisture in that order.

#### 3.3.2. Oversampling Ratios

**Figure 5:**
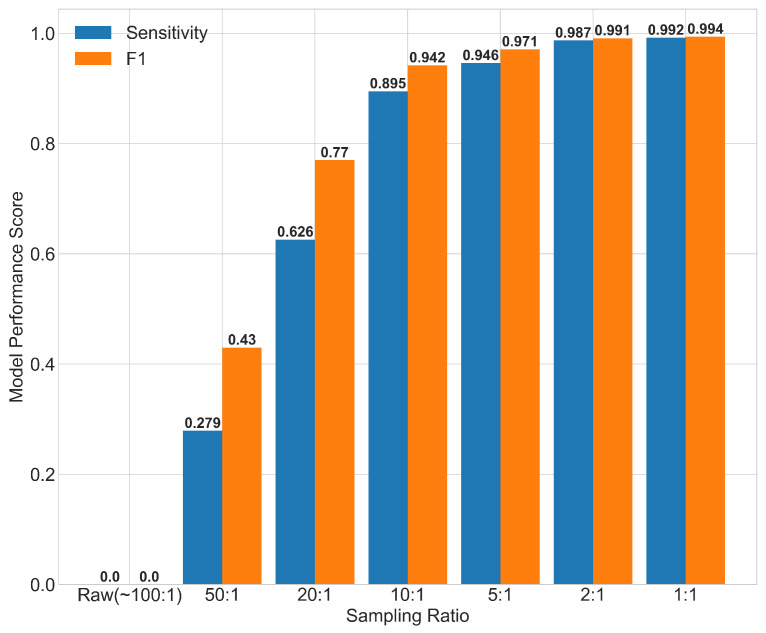
Performance metrics for the random forest model trained and run using datasets with different oversampling ratios (sampling ration are shown as non-outbreaks: outbreaks). Oversampling was performed based on the Synthetic Minority Oversampling Technique (SMOTE) in the pre-processing stage (see Section 2.3).

To assess the model sensitivity to the imbalanced training dataset input, different oversampling ratios were considered using SMOTE oversampling (Figure 5). While a 1:1 ratio produced the best accuracy metrics (sensitivity = 0.992, F1 score = 0.994), having a perfectly balanced dataset would be unrepresentative of the cholera disease incidence in a real-world scenario. Although the most realistic scenario is difficult to establish due to common issues of under-reporting cholera outbreaks, the sampling ratio of 1:10 (one outbreak for every ten non-outbreak data points), which was considered more plausible in coastal endemic areas of India, produced promising results (sensitivity = 0.895, F1 score = 0.942).

#### 3.3.3. Machine Learning Method (ML)

**Figure 6:**
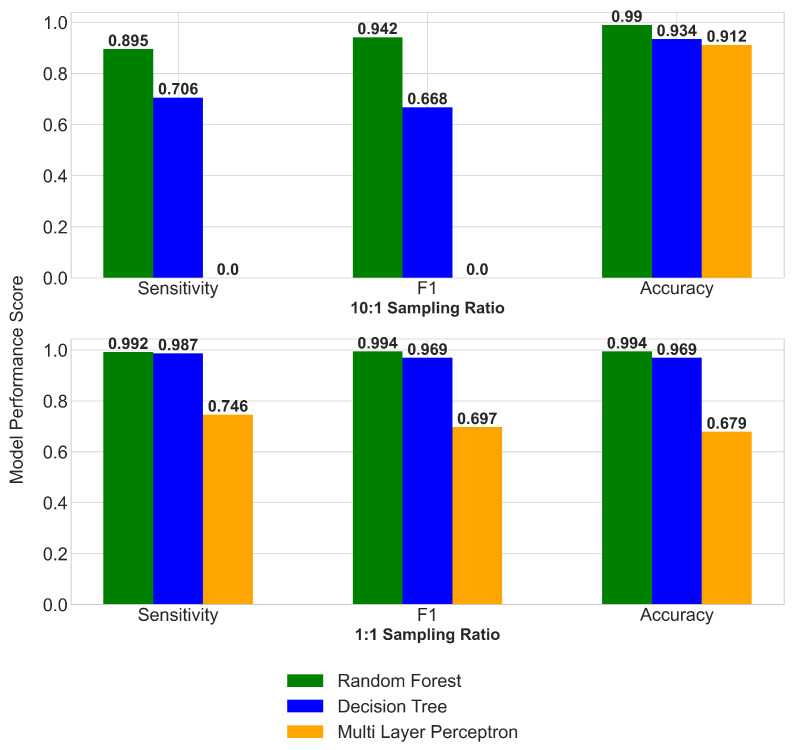
Sensitivity analyses of the choice of machine learning methods for two sampling ratios. Performance metrics are shown for the Random Forest classifier, a single decision tree model and a multi-layer perceptron neural network model generated using the same training and test datasets. Performance metrics were obtained based on a 10:1 sampling ratio (upper panel) and a 1:1 sampling ratio (lower panel).

To explore the model sensitivity to the choice of the machine learning technique, we performed the analysis using two other Machine Learning (ML) methods: a single Decision Tree and a Neural Network (Multi-Layer Perceptron). These methods were selected because they offered different levels of transparency and complexity, yet retaining an ability to deal with complex, non-linear relationships. Specifically, we chose a deep neural network to explore a more black-box method that creates its own features compared to the RF classifier which is based on the data available, and a single decision tree to explore the benefits of the RF classifier averaging across multiple decision trees. To compare the results from the different ML methods, we used the same training dataset and the same selection of ECVs, lagged values and encoders as we did for the random forest classifier (Figure 6). When the analyses were performed using a 10:1 oversampling ratio (as used for our final RF model), the multi-layer perceptron neural network was not able to detect any outbreaks, resulting in a null score for the performance metrics of sensitivity and F1 Score. The RF classifier, utilizing multiple estimators, performed stronger than the single Decision Tree, with sensitivity increasing from 0.706 to 0.895 and F1 Score increasing from 0.668 to 0.942 with the introduction of the ensemble learning method used in the RF classifier (Figure 6 upper panel). When a 1:1 oversampling ratio was used, the multi-layer perceptron was able to detect cholera outbreaks, but still fell short of the decision tree and RF classifier. The accuracy metric results obtained with the RF classifier were all above 0.99 (Figure 6 lower panel), whilst the multi-layer perceptron produced results similar to those seen by the other methods at the 10:1 sampling ratio.

## 4. Discussion

### 4.1. Usage of Remotely-Sensed ECVs for Cholera-Outbreak Risk Analyses

This study presents a novel ML application analyzing multiple ECV datasets in combination, to explore a range of contributing environmental factors and their influence on cholera outbreak risk. The consistent, long-term time series of remotely-sensed data from ESA-CCI were ample to provide contextual climate information for nine years of cholera outbreak data and explore application across seasons and locations (Appendix A Figure A1). However, whilst investigating the influence of ECVs on cholera outbreak risk, we also recognized a limitation of ML techniques, which is the difficulties in interrogating the thresholds and relationships with each individual input variable. In fact, a longstanding caveat of ML is that, as it has to learn and generate predictions from complex relationships, it becomes difficult to make the black box mechanisms more transparent for scientific value.

An important development stage of an ML model utilizing a cross-ECV approach is the feature selection, and particularly the need to identify variables displaying collinearity, as they may be contributing similar information towards the model (e.g, [62]). In the present work, a high correlation is to be expected among ECVs related to the water cycle. We found that the three ECVs that did not reduce accuracy when permuted were sea surface temperature, soil moisture and precipitation (Appendix A Figure A2). The latter pair, and their respective lags, were significantly positively correlated (Spearman’s Rank Correlation *p*-value < 0.05). These two variables were initially included in our analyses, as previous studies have found changes in rainfall patterns and especially drought conditions to be associated with early spring outbreaks, while flood volumes and extent were shown to be associated with autumn outbreaks, suggesting complex seasonal variations in these variable’s relationships with cholera incidence in the Bengal Delta [31]. Individual testing of ECVs showed that soil moisture contributed more strongly than precipitation towards improving the model performance, possibly indicating that soil moisture may be a more direct proxy of environmental-to-human contamination associated with changes in water availability as suggested in previous reports [16].

Sea surface temperature has been found to influence cholera variability from warming waters increasing moisture convergence and subsequently precipitation [23]. Our analyses showed that sea surface temperature in the current month or with lags of up to two months before a cholera outbreak did not improve the performance of the model. While some studies have reported sea surface temperature to be correlated with cholera incidence in different regions of the world (e.g., [24,63]), in the case of the Bengal delta region, the correlation has been demonstrated with large lagged effects from sea surface temperature anomalies in the previous winter [23,31]. A further example where sea surface temperature has been reported to show collinearity with another ECV, is from the previous study by [5], which found a positive relationship between sea surface temperature and chlorophyll-a concentration. The authors suggested that increases in sea surface temperatures may stimulate phytoplankton growth which in turn enhance can host and nutrient source availability for *Vibrio* species. Therefore, based on our model performance analyses and previous studies of collinearity, the variable soil moisture was retained (over sea surface temperature and precipitation) to ensure that *V. cholerae* environmental suitability could be considered without information duplication or over-fitting.

### 4.2. Machine Learning Techniques for Cholera Risk

Amongst the different machine learning techniques tested in this study, the RF classifier was the most effective, performing the best with a 10:1 oversampling ratio and overall being capable of overcoming the issue of imbalanced data, unlike the multi-layer perceptron and the single decision tree which required more balanced samples. Although we have begun to compare the sensitivity of models’ performance based on different machine learning techniques, we acknowledge that far more techniques could be investigated, however, such analyses are beyond the scope of this study.

Our RF classifier for coastal regions in India achieved a sensitivity of 0.895 after oversampling, which lies in a higher range when compared to a previous study that tested an RF classifier on imbalanced cholera data in Tanzania, and which was reported to achieve a sensitivity of 0.645 after oversampling and Principal Component Analysis [35]. This difference in sensitivity may be attributed to the different datasets in terms of a geographically distinct location, another cholera surveillance program and our study implementing a wider and more diverse range of ECVs, including oceanic ones, while [35] focused on land variables; land surface temperature metrics, rainfall, humidity and wind. Interestingly, the latter study also explored other ML techniques and found an XGBoosting algorithm to perform better than their RF classifier for cholera incidence in Tanzania, using scaling parameters to focus on sensitivity, providing scope to explore different machine learning methods in the future, and particularly how some might be more applicable to different study locations or datasets.

Ultimately important, the choice of ML technique and method improvements were based on the RF classifier’s ability to function in the conditions of endemic cholera patterns reported in the input datasets. A balanced sample might increase the robustness of the model to detect single outbreaks reported at the individual district level in the test dataset, and improve the sensitivity in the districts reporting fewer cholera outbreaks. Previous studies have indicated that cholera might be under-reported to a factor of six [64], suggesting that if all cholera outbreaks were reported, the sample might be less imbalanced which could help to improve model accuracy. However, this would not be a true reflection of the present input dataset which was largely imbalanced (see Section 2.3) and further model improvement would need to consider increasing the robustness and sensitivity of the model to detect even single outbreaks, while using imbalanced samples. For datasets spanning a longer time period and for which a sufficient number of cholera outbreaks are available, improvements on the model methods could involve using an ensemble of cross-validation subsets to help to justify the model robustness or imposing a cost penalty on the minority class misclassification to help the RF model learning from very imbalanced data [65,66,67]. Furthermore, future conceptual developments of the model might consider incorporating socioeconomic data, extreme weather anomalies, as well as considering the use of higher spatio-temporal resolution ECV datasets [68] that could support detection of micro-niches of *V. cholerae* habitat suitability.

### 4.3. RF Model Feature Performance Analyses

Despite large variations in monsoon-related hydroclimatic conditions between the Arabian Sea and Bay of Bengal, the model performance showed no apparent difference in either West and East coastal districts of India. This is likely due to the location encoder, which ranked the highest in terms of feature importance (Appendix A Figure A3). The East and West location encoder causes the RF model to split the decision trees at an early stage and allows the different hydroclimatic responses to be dealt with separately in the prediction phase.

The one-month and two-month lagged values of chlorophyll-a were identified as second and fourth strongest contributors to the RF model cholera prediction, respectively. Chlorophyll-a concentration is the key variable to estimate primary production in the marine environment [69], and cholera outbreaks have previously been found to occur following plankton blooms [25]. Phytoplankton blooms can stimulate bacterial growth by providing a food source, altering the pH of the water, offering host protection and supporting the growth of zooplankton and other marine hosts and reservoirs of *V. cholerae* [1,2,3,4,16,24,28,70]. Lagged effects are to be expected as the breakdown of phytoplankton increases nutrient availability for *V. cholerae* to flourish [16,71], and is in agreement with previous studies showing the influence of plankton on cholera to be present up to a lag of eight weeks [72].

The third most important feature highlighted was sea surface salinity of the month of the outbreak. To our knowledge, this represents the first inclusion of remotely-sensed salinity data in a machine learning application for cholera risk, and as such, our model fulfils a gap in the existing literature. Previous work on the relationships between salinity and *V. cholerae* has been based on laboratory experiments [25] and field sampling [27,73], which showed that the bacteria are able to tolerate moderate salinity as well as higher concentrations, with the most favorable salinity being found for brackish and coastal waters. The dilution effects of monsoonal rains have also been shown to favour the replacement of the classical strain by the El Tor strain due to its increased relative fitness under reduced salinity [29]. Our study demonstrates the influence of salinity on cholera outbreaks on a synoptic scale using remotely-sensed observations, and the importance of this variable as highlighted here underpins further usage to improve surveillance and forecast of clinical risk of *Vibrio* infections in coastal systems. In future studies, long-term continuous observations of sea surface salinity at high spatial resolution (4 km or higher), especially in coastal areas, would be extremely valuable as the risks of cholera or vibriosis are predominantly associated with human exposure (bathing, recreational waters) and seafood consumption occurring close to the shore.

The land surface temperature of the month of the outbreak was identified as the fifth most important feature, and had the strongest performance when the model was run using only land surface temperature variables alongside the location and season encoder (Figure 4). Previous studies have highlighted the important relationships between cholera incidence and land surface temperature. For instance, land surface temperature can increase the water temperature of shallower water bodies such as estuaries, which has been associated with an increase in cholera outbreaks in Bangladesh [74]. In East Africa, a one-degree increase in temperature has been found to double the risk of a cholera outbreak during the rainy seasons, particularly in areas without safe water or sanitation [75]. However, while cholera risk has been related to high air temperature with two-month lag (followed by high precipitation) [32], our study also found that the land surface temperature in the current month is an important feature, in addition to the lagged effects which can also be used to predict cholera outbreaks.

Finally, sea level anomalies, while not appearing amongst the top-five variables in the feature importance analyses, still contributed to improving the model performance, as permuting the variable led to a reduction in model accuracy (Figure 4). The relationships between sea level anomalies and cholera have been well established using remote sensing [24,26] based on saltwater and plankton intrusions into inland waters, increasing the suitability for *V. cholerae* in brackish waters [28]. Specifically, the years with the highest peaks in sea level were found to coincide with the years with the highest cholera incidence [26]. It is noteworthy that the yearly average used in the latter study is distinct in comparison to our monthly analysis, and it may be useful to explore further the importance of the choice of temporal resolution in sea level anomalies as well as in other ECVs ([22]).

### 4.4. Study Limitations and Opportunities

#### 4.4.1. Metrics of Model Accuracy

In the present study, the variations observed in the RF model accuracy across the individual districts are likely to be influenced by the quality of the input cholera dataset for each region, which in turn reflect the strength of health infrastructure in each region. It is noteworthy that the districts and states in which the model performs the most accurately have also been identified as those which benefit from the best healthcare systems, increasing the accuracy of the training data and consequently the model results. The Health Index scores from the ‘Healthy States Progressive India’ Government Report finds Kerala and Maharashtra as the top first and third district respectively, alongside Tamil Nadu in being labelled as ‘front runners’ in terms of overall performance of health systems and service delivery [76]. This could partially explain why the model has the strongest performances in these states (sensitivity > 0.90) due to the effective reporting infrastructure, and strong health systems which may reduce the non-environmental confounding factors mentioned previously. Additionally, the way in which the accuracy metric is calculated may lead to some discrepancy. For instance, Balasore district in Odisha State has an accuracy score of 0.962, but the one outbreak in the test dataset was a false negative, and hence the accuracy score reflects the 25 non-outbreaks it correctly identified. Such results provide an impetus for utilizing multiple evaluation metrics in combination, to provide a more harmonic insight into model performance. In the application to cholera-outbreak risk, false negatives could have disastrous consequences, as a cholera outbreak would be missed in the forecast and communities would not be able to prepare. Therefore, a surplus of false negatives is of high concern, giving greater weighting to the metric of sensitivity. Conversely, a surplus of false positives could indicate under-reporting of cholera outbreaks in a region and could be seen as over-cautious reporting. Furthermore, false positives could trigger false warning announcements and over-preparedness leading to extra costs and demands on often limited health resources and eventually cause the loss of trust in the cholera-risk forecast by the local populations.

#### 4.4.2. Epidemiological Records of Cholera Outbreaks

Previous studies that have utilized the IDSP weekly reports of disease outbreaks have noted their outstanding importance for epidemiological research on cholera but also noted some limitations, particularly along with the issue of under-reporting of cholera cases in India [77]. Under-reporting and data gaps have previously been linked with a lack of laboratory capacity and funds, weak engagement of private referral laboratories, lack of understanding of case definitions, or patients not seeking hospital treatment if symptoms are mild, with these cases then falling beneath the health surveillance radar [64,78,79,80], as well as a lack of availability of rapid diagnostic cholera tests leading to many cholera cases reported as ‘acute diarrhoeal disease’ [64,77]. Patterns of under-reporting could contribute towards the presence of false negatives in the RF model results, due to an incomplete training dataset. Furthermore, [81] highlights the issue that cholera cases tend to be recorded from outbreaks, omitting sporadic cases, and that states with better reporting systems are likely to record more outbreaks. This is not an issue unique to India, with for instance Leo et al. [35] reporting issues with cholera dataset quality in Tanzania. Additionally, data collection bias and over-reporting might occur during outbreaks when cholera cases are commonly reported without laboratory confirmation, based on clinical signs and symptoms alone [82]. Efforts towards improving the IDSP system to reduce non-uniform reporting per state in India are on-going [79], and previous studies [77,81], as well as the novel ML application, demonstrated in the present study, lend strong support to the crucial need to sustain and strengthen national cholera surveillance systems to improve our understanding of environmental cholera risk.

#### 4.4.3. Socio-Economic Conditions and Seasonal Extremes

While the RF model shows the highest sensitivity score (0.933) to cholera outbreaks reported during pre-monsoon, the sensitivity score in monsoon is found to be lower (0.857). The highest sensitivity score achieved in pre-monsoon may be related to the dynamic of cholera outbreaks reported in that season which may be more likely driven primarily by environmental factors, whereas cholera outbreaks reported in monsoon and post-monsoon months may be more difficult to predict due to other confounding factors. For instance, a previous study has attributed surges of cholera in Kolkata in monsoon and post-monsoon periods, particularly in August 2015, to water-logging and drain overflow from monsoonal precipitation, contaminating drinking water sources in the month before the outbreaks [83]. Under more extreme conditions, variations in cholera incidence may be confounded by socioeconomic factors including hygiene practices, access to safe drinking water, availability of sanitation and drainage systems, which the monsoon heavy rainfall can make more vulnerable and exacerbate the burden of cholera [32,84]. The results of the RF model highlight that, in endemic areas, while environmental variables alone are still largely the driving force behind cholera outbreaks [32], there is still a number of cholera outbreaks missed, suggesting room for improvements.

The effects of extreme seasonal perturbations on socio-economic conditions present a key challenge for cholera prediction models in both endemic and epidemic regions. To provide robust warning systems to vulnerable populations in the case of natural hazards, models need to incorporate transmission mechanisms associated with water and sanitation infrastructure alongside environmental variables. The present findings concur with [1] stating that sanitary conditions should not be ignored. Future studies need to consider enriching their models and analysis with a combination of both environmental and socioeconomic data [85].

## 5. Conclusions

This study has demonstrated a novel application of the effectiveness of random forest classifiers as a machine learning technique for cholera risk applications based on an analysis of seven remotely-sensed ECVs and their respective lagged values. In particular, we showed that the variables contributing most strongly to the RF model cholera prediction included the one-month and two-month lagged values of chlorophyll-a concentration, sea surface salinity, land surface temperature and sea level anomalies, in order of their contribution strength to the model performance results respectively. Furthermore, it is noteworthy that while previous studies have utilized ECVs, this study demonstrates the first usage of remotely-sensed sea surface salinity data in machine learning analyses of a combination of ECVs to detect the risk of cholera outbreaks.

The model showed promising results when tested on individual districts in coastal India, underlining its potential to perform accurately across a country with large climatological differences evident spatially. The model performed better in areas with outbreaks reported routinely or annually, but was less effective in areas where there are fewer cholera outbreaks to both train and test the model. These findings suggest that the model would be more suited for detecting cholera outbreaks in endemic areas but might be less likely to detect more sporadic, epidemic cholera events. The latter are more likely to occur due to an import by a contaminated traveller, and/or following a natural disaster or civil disorder which would affect living conditions [86,87].

In the future, the present novel analyses may be refined and the model continued to be tested using different cholera surveillance datasets and in diverse locations, to improve and broaden its applicability in other cholera endemic regions. Further model developments, accounting for socio-economic conditions, are anticipated to help us to improve model performance in seasons affected by confounding factors, such as during the monsoon when intense rainfalls place high pressure on populations’ access to safe drinking water, and availability of sanitation and drainage systems [84]. Future efforts would also need be directed in supporting long-term, continuous, accurate ECV observations in coastal areas and cholera surveillance programs, which are crucial for model validation, and to improve mechanistic understanding of environmental processes influencing cholera outbreaks.

## Figures and Tables

**Table 1:** Essential climate variable datasets used in the present study.

Dataset	Variable	Temporal Resolution	Spatial Resolution	Period	Reference	Source
CCI SST	Sea Surface Temperature (K)	Monthly	0.05°	2010–2018	[39]	climate.esa.int
CCI Sea Surface Salinity	Sea Surface Salinity (psu)	15 days	25 km	2010–2018	[40]	climate.esa.int
CCI Sea Level	Sea Level Anomaly (m)	Monthly	0.25°	2010–2015	[41]	climate.esa.int
CCI Ocean Colour	Chlorophyll-A Concentration (mg/m${}^{3}$)	Monthly	4 km	2010–2018	[42]	climate.esa.int
CCI Soil Moisture	Soil Moisture combined product (m${}^{3}$/m${}^{-3}$)	Daily	0.25°	2010–2018	[43,44,45]	climate.esa.int
CCI Land Surface Temperature	Average Day Land Surface Temperature (K)	Daily	0.05°	2010–2018	[46]	climate.esa.int
ERA Interim	Synoptic Means of Total Precipitation (m)	Monthly	0.75°	2010–2018	[47]	cds.climate.copernicus.eu
AVISO Altimetry	Sea Level Anomaly (m)	Monthly	0.25°	2016–2018	AVISO+	aviso.altimetry.fr

**Table 2:** Random Forest model performance metrics on test data for each season for coastal districts in India.

Test Data	Sensitivity	F1 Score	Accuracy
**All seasons**	0.895	0.942	0.990
**Winter (JF)**	0.868	0.930	0.991
**Pre-monsoon (MAM)**	0.933	0.960	0.991
**Monsoon (JJAS)**	0.857	0.923	0.986
**Post-monsoon (OND)**	0.886	0.939	0.994

## Abbreviations

The following abbreviations are used in this manuscript:

1 m	1 month lagged value
2 m	2 month lagged value
CCI	Climate Change Initiative
Chlora	Chlorophyll-a Concentration
ECV	Essential Climate Variable
ESA	European Space Agency
IDSP	Integrated Disease Surveillance Programme
LST	Land Surface Temperature
ML	Machine Learning
PDF	Portable Document Format
Precip	Total Precipitation
RF	Random Forest
SLA	Sea Level Anomaly
SM	Soil Moisture
SSS	Sea Surface Salinity
SST	Sea Surface Temperature
